# Awareness, Experience, and Knowledge of Farming Households in Rural Bangladesh Regarding Mold Contamination of Food Crops: A Cross-Sectional Study

**DOI:** 10.3390/ijerph181910335

**Published:** 2021-09-30

**Authors:** Nicholas N. A. Kyei, Jillian L. Waid, Nurshad Ali, Sabine Gabrysch

**Affiliations:** 1 Heidelberg Institute of Global Health, Heidelberg University, 69120 Heidelberg, Germany; jillian.waid@pik-potsdam.de (J.L.W.); sabine.gabrysch@charite.de (S.G.); 2 Research Department 2, Potsdam Institute for Climate Impact Research (PIK), 14412 Potsdam, Germany; 3 Institute of Public Health, Charité—Universitätsmedizin Berlin, 10117 Berlin, Germany; 4 Department of Biochemistry and Molecular Biology, Shahjalal University of Science and Technology, Sylhet 3114, Bangladesh; nur_rubd@yahoo.com

**Keywords:** agriculture, KAP survey, mycotoxins, food spoilage, south-east Asia

## Abstract

Aside from specific environmental conditions, poor agricultural practices contribute to mold and thus the mycotoxin contamination of crops. This study investigated Bangladeshi farming households’ (i) awareness of and experience with mold contamination of food crops; (ii) knowledge and awareness of the timing, causes, and consequences of mold and mycotoxin contamination; and (iii) knowledge of the recommended agricultural practices for controlling and preventing mold contamination of food crops. A survey was conducted with 1280 households in rural areas of Habiganj district, Bangladesh. Basic descriptive statistics were calculated, and mixed-effects linear regression analyses were performed to examine associations between household characteristics and overall knowledge scores. The awareness of mold contamination of food crops was very high (99%; 95% CI: 98–100%) and a shared experience among households (85%; 95% CI: 80–88%). Yet, the majority (80%; 95% CI: 76–84%) demonstrated a low level of knowledge of the timing, causes, and preventive practices regarding mold contamination of crops. Knowledge scores were similar over demographic groups and better for households with more arable land. The findings suggest a generally insufficient knowledge of the conditions that favor mold contamination and the measures for preventing mold contamination of food crops. These findings underline the need for tailored interventions to promote good agricultural practices and reduce mold contamination of food crops.

## 1. Introduction

Mold contamination of food crops is widespread and constitutes a leading global food safety concern [1]. The growth and metabolism of mold does not only result in agricultural and economic losses through food spoilage but also has severe consequences for both human and animal health through associated mycotoxin production [2,3,4,5,6,7,8,9]. Mycotoxins refer to a diverse group of chemical compounds produced as secondary metabolites of mold, which are toxic to humans and animals in low concentrations [10,11]. Food crops, including human dietary staples such as wheat, maize, rice, and other cereal grains, are prone to mold contamination before harvesting, during harvesting, and during post-harvest handling and storage.

Mycotoxin occurrence at levels above European Union and Codex limits is estimated to affect around 20% of food crops globally, while the occurrence above detectable levels ranges between 60% and 80% [12]. Mycotoxins are resistant to several processing and cooking practices [13,14]. Consequently, they are a ubiquitous exposure risk, especially in low-income settings, where populations eat monotonous diets of frequently contaminated staple crops [1].

In general, the mold contamination of food crops and subsequent mycotoxin production depends on several factors, including the plant species; environmental conditions, such as moisture and temperature; and the presence of other fungi, microbes, and insect pests [15,16,17]. Besides these factors, which are usually not under the control of farmers, certain agricultural practices influence the mold contamination of crops and subsequent mycotoxin production [18,19,20,21]. Although mycotoxins cannot be eliminated from food or feed supplies, their levels can be substantially reduced by adopting the recommended agricultural and management practices [18,21].

In Bangladesh, for instance, several biomarker surveys [22,23,24,25,26,27,28] suggest a frequent dietary exposure to multiple mycotoxins among both rural and urban populations. Despite the availability of detailed codes of practice for preventing and reducing the mycotoxin contamination of cereals at every stage of crop management [29], there is limited information on the level of knowledge, awareness, and practices (KAP) of farmers regarding mold contamination of food crops and the application of the recommended guidelines, especially in low-income settings with a high occurrence of mycotoxins. As commercial farmers are often expected to fulfill specific standards, they most likely know more about these codes of practice than subsistence farmers. In low-income settings with less commercial agriculture, promoting these evidence-based and positive agricultural practices among rural households involved in subsistence farming could help to minimize such harmful exposure.

Therefore, this study aimed to investigate farming households’ (i) level of awareness and experience with mold contamination of food crops; (ii) knowledge and awareness about the timing, causes, and consequences of mold and mycotoxin contamination; and (iii) knowledge of the recommended agricultural practices for controlling and preventing mold contamination of food crops.

## 2. Materials and Methods

### 2.1. Study Population

This study was conducted within the *Food and Agricultural Approaches to Reducing Malnutrition* (FAARM) cluster-randomized controlled trial in two sub-districts of Habiganj district in Bangladesh’s Sylhet Division (ClinicalTrials.gov ID: NCT025-05711). FAARM included 2700 young married women in 96 settlements (geographic clusters) who were interested in gardening and had access to at least 40 m^2^ of land. Settlements were randomized into 48 intervention and 48 control clusters. The FAARM trial evaluated the impact of a homestead food production program, implemented by the international non-governmental organization Helen Keller International, on undernutrition in young children. Alongside nutrition and hygiene education, the FAARM project promoted homestead production of nutritious foods through home gardening and poultry rearing among intervention households to improve garden productivity and dietary diversity and to eventually reduce child stunting. Further information on the FAARM trial is available in the study protocol [30]. The *Maternal Exposure to Mycotoxins and Adverse Pregnancy Outcomes* (MEMAPO) prospective cohort study is an add-on study to the FAARM trial that collected data on a subsample of 447 pregnant FAARM women to investigate the role of mycotoxins in the development of specific adverse pregnancy outcomes.

### 2.2. Questionnaire Preparation

A structured questionnaire was developed collaboratively with local experts and farmers, adapting recommended guidelines for preventing mold contamination of cereals [29] to the local context. Based on pre-test feedback, further revisions were made, and a visual aid with a picture of moldy items (bread, orange, and a wall) was prepared to help explain mold to participants where needed. The final survey instrument included a total of 15 questions (Supplementary Table S1a).

### 2.3. Study and Sampling Design

A cross-sectional study was conducted among a sample of FAARM participants during the FAARM endline household survey from November 2019 to April 2020. As awareness of mold appeared high among FAARM respondents during the first two weeks of data collection, instead of asking all households, a sub-sample was targeted to enable faster data collection while maintaining sufficient power. We examine data collected from all FAARM households visited during the first 16 days of data collection, FAARM households containing pregnant women who had been recruited in the MEMAPO study, and an additional random 25% of the remaining FAARM households. The sub-sample was drawn from all FAARM settlements. Overall, a total of 1371 households were targeted for inclusion in this study.

### 2.4. Socio-Demographic Characteristics

To characterize the recruited households, data on the following variables were extracted from the FAARM endline survey and illustrated in Table 1: religion, household size, ownership of livestock or poultry, size of homestead and agricultural land, household wealth quintile, and household main income sources, as well as age, sex and educational level of the head of the household. The main income source of each household was identified for the primary income earner. According to Equity Tool guidelines, each household’s position within the 2014 Demographic and Health Survey national wealth quintiles was calculated from household asset information [31,32].

### 2.5. Knowledge Assessment

The survey instrument collected the following specific knowledge information: (i) awareness of mold contamination of food crops; (ii) the stages of crop production where mold contamination can occur; (iii) conditions and practices that promote mold contamination during growing, harvesting, and storage; (iv) the consequences of mold contamination of crops; (v) awareness of mycotoxin production; (vi) stability of mycotoxins to cooking and food/feed preparation, (vii) harmful effects of mycotoxin exposure in farm animals; (viii) harmful effects of mycotoxin exposure in humans; and (ix) recommended measures for preventing mold contamination at all stages of crop production [29]. This information was summarized and presented in three knowledge categories: (a) timing of mold contamination and favorable conditions, (b) mycotoxin production and harmful effects, and (c) preventive practices. For knowledge assessment, a correct answer to any question was given as a score of one; an incorrect answer or answer of “no” was given as a score of zero. An overall knowledge score was computed as the sum of all correct responses in each knowledge category. The maximum attainable score for perfect knowledge in all areas was 81 points: 29 points (36% of total score) for knowledge on the timing of mold contamination and favorable conditions, 21 points (26% of total score) for knowledge of mycotoxin production, and harmful effects, and 31 points (38% of total score) for knowledge of preventive practices. These percentages indicated the relative weights or importance given to knowledge in the three knowledge areas. For descriptive analysis, the knowledge scores were classified as follows: less than 30% of the maximum attainable score in each knowledge category, as well as the overall score, was classified as “low knowledge”, between 30% and 59% of the maximum attainable scores as “fair knowledge”, and 60% or more of the maximum possible scores as “good knowledge”. Supplementary Table S1b provides details of the survey questions and the knowledge scoring criteria.

### 2.6. Data Collection

As the terms “mold” and “mycotoxin” are technical, respondents were first screened for their awareness and understanding of identified local terms for mold and mycotoxins (“Chhatrak” or “Chita” or” Maista” or “Maiska” or “Kalo Dag”). To those who were unsure or gave an inaccurate definition, these terms’ proper meanings were explained in the local language with the help of the visual aid. Thereafter, only those who indicated that they knew about mold were further interviewed on their level of awareness, experience, and knowledge regarding mold contamination of food crops. Where respondents did not know the answer to a primary question, related sub-questions were assumed to be also unknown and thus skipped. Trained data collection officers administered the questionnaires through face-to-face interviews with respondents without reading out answer options but by selecting mentioned options and entering any other answers provided. After 25 March 2020, the last few interviews with 31 households were conducted by phone due to the COVID-19 pandemic. All data were collected and entered electronically using Open Data Kit on tablets [33] as outlined in the FAARM protocol [30]. Overall, data were obtained for 1280 of the 1371 targeted households (93%) and 429 of the 447 targeted MEMAPO households (96%).

### 2.7. Statistical Analysis

Descriptive statistics (e.g., proportions and means) were calculated using sampling probability weights to reflect the unequal probabilities in selecting study participants due to the study’s sampling design and controlling for clustering, i.e., similarity of participants at the settlement level. Wald tests were used to examine differences in the response proportions and average knowledge scores between FAARM intervention and control households. Linear regression models with both fixed and random effects (mixed-effects linear regression), applying sampling probability weights, and controlling for settlement-level clustering, were used to examine associations between household socio-demographic characteristics and mold mycotoxin knowledge score. Subsequently, a multivariable mixed-effect model was built with those socio-demographic variables associated in the crude analysis (*p* < 0.15), adjusting a priori for household wealth and the household head’s educational level. For ease of interpretation, household size and size of the homestead and agricultural land were categorized as illustrated in Table 2 and Supplementary Table S2, informed by categories used in previous research in Bangladesh [34]. Data management and analysis were performed with Stata version 14.2 SE (StataCorp LLC, College Station, TX, USA).

## 3. Results

### 3.1. Socio-Demographic Characteristics of the Study Population

The characteristics of the surveyed population are shown in Table 1. Nearly three-quarters of households were Muslim, with the remaining being Hindu. At the time of the FAARM endline survey (November 2019 to April 2020), about half of the households belonged to the upper two wealth quintiles of the 2014 Bangladesh Demographic and Health Survey wealth index, while more than a third belonged to the middle and a tenth to the lower two wealth quintiles. On average, household heads were in their early-to-mid forties, and nearly all of them (96%) were male. Only 6% of household heads had completed secondary school or higher degrees, while 41% had no formal education. The households were involved in subsistence farming with some livestock or poultry rearing. They therefore had various income sources, with about 28% relying mainly on rice farming and an additional 5% on other crops. Four out of five households also reared poultry, with or without other livestock.

### 3.2. Level of Awareness and Experience with Mold Contamination of Crops

The level of awareness and experience of the surveyed population with mold contamination is summarized in Table 3. Overall, 60% of respondents spontaneously recognized the local terms for mold that we used. Most of the respondents who recognized the terms (84%) could also correctly explain or define mold. After explaining what was meant by mold with the help of the visual aid to those who appeared unfamiliar with the terms, all respondents claimed to know about mold, almost all (99%) were aware that mold could contaminate food crops, and most (85%) had personally experienced such contamination. Of these, the vast majority (82%) reported experiencing mold contamination before harvesting, with many fewer people reporting mold contamination during harvesting (17%) and at the post-harvest stage (26%). More respondents from FAARM intervention households than respondents from control households could correctly explain the local mold term (58% vs. 44%). While mold contamination of food crops before harvesting was a common experience, FAARM intervention households reported this somewhat less frequently than the control households (65% vs. 75%).

### 3.3. Knowledge of Timing, Causes, and Consequences of Mold and Mycotoxin Contamination

**Figure 1:**
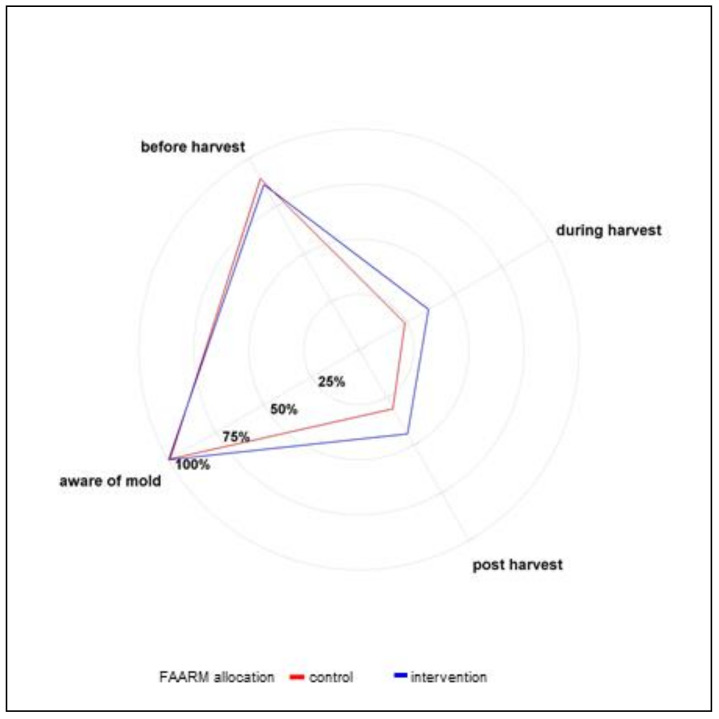
Awareness and timing of mold contamination (*n* = 1280).

**Figure 2:**
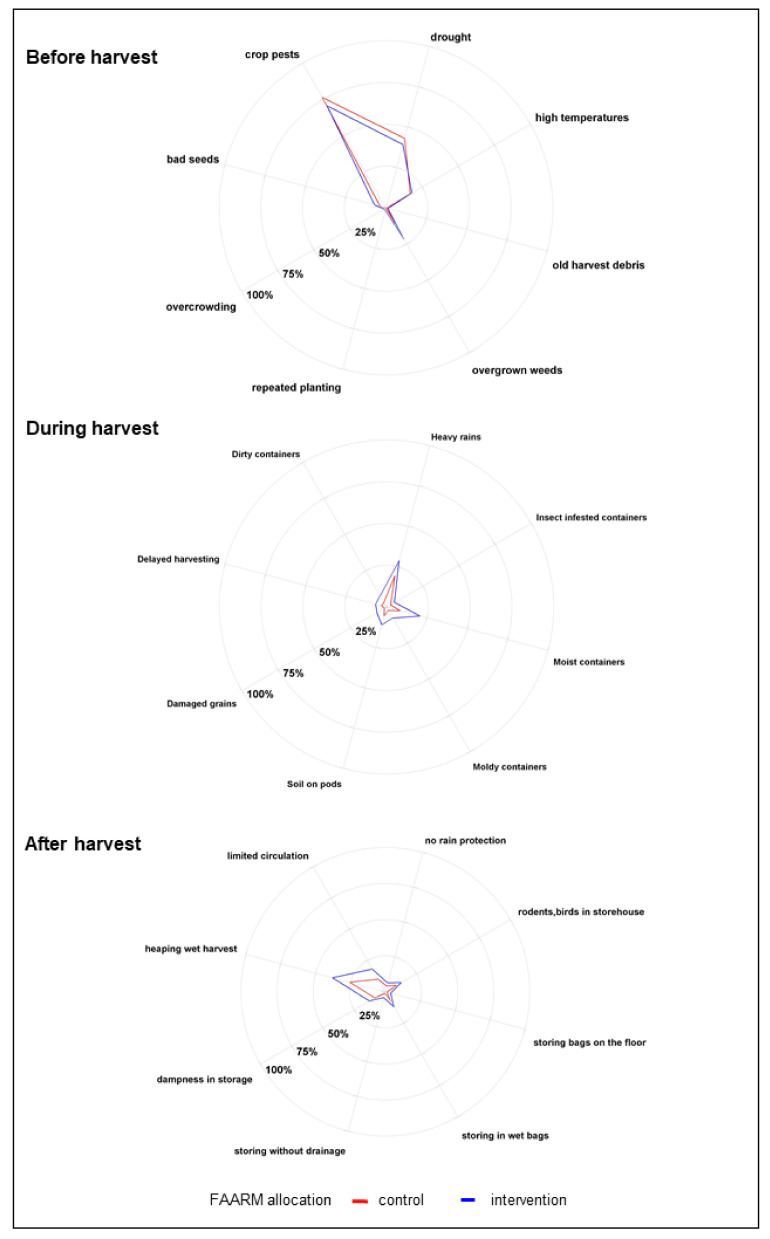
Conditions for mold proliferation (*n* = 1280).

**Figure 3:**
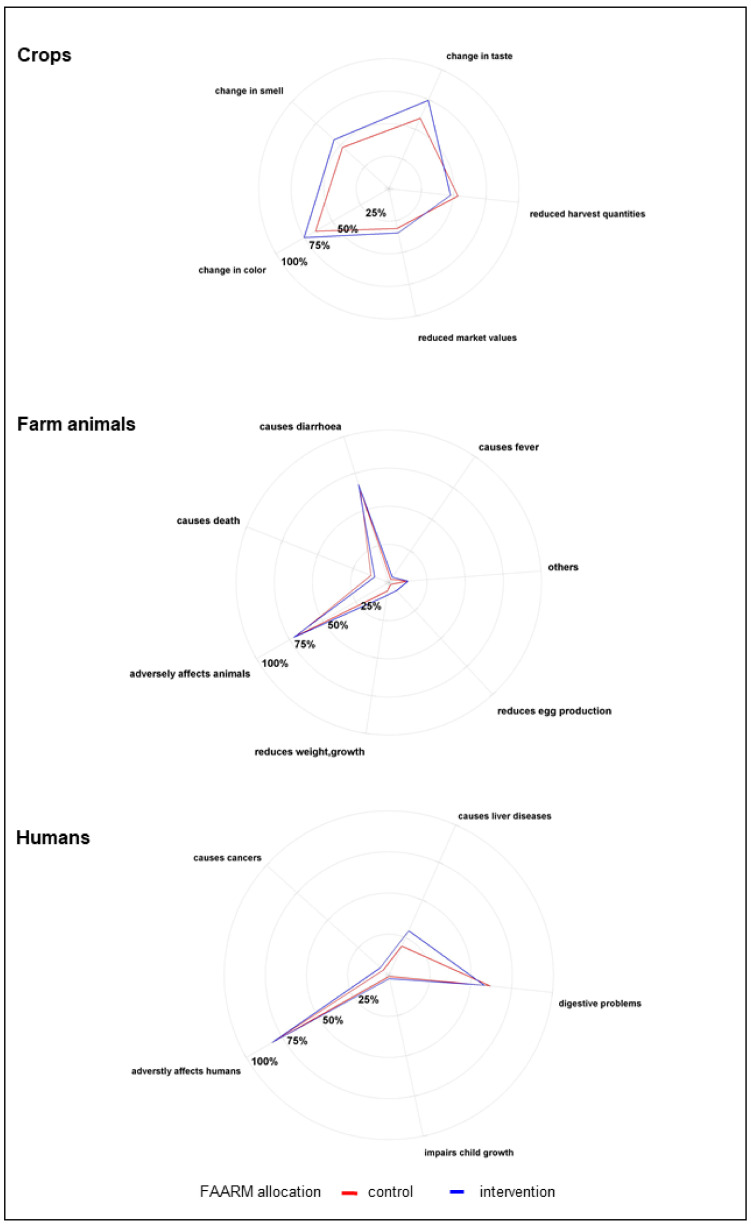
Harmful effects of mold contamination (*n* = 1280).

The responses to the knowledge questions on the contamination conditions, the harmful effects of mold contamination, and preventive practices are summarized in Table 4 and Figure 1, Figure 2, Figure 3. In line with their reported experience, the vast majority of respondents (88%) knew that mold contamination of food crops could occur before harvesting, whereas 31% and 38% knew that contamination could occur during the harvesting and post-harvesting stages, respectively. Comparatively, FAARM intervention households were more aware of potential contamination during the harvesting and post-harvest stages.

Among the respondents aware of the possibility of mold contamination before harvesting, the presence of insects/pests was the predominantly mentioned condition favoring mold proliferation (83%). About half of respondents knew that drought conditions could favor mold proliferation before harvest, whereas about one-fifth mentioned high temperatures and overgrown weeds as causes of mold proliferation. Among the respondents who were aware of mold contamination during harvesting, heavy rain during harvesting was the predominantly known condition for mold proliferation (79%). Around half of the respondents correctly mentioned the use of moist containers as a risk factor for mold during harvesting, while around a quarter mentioned leaving soil on pods. Among respondents aware of mold contamination during the post-harvest and storage stage, heaping wet, freshly harvested crops was the predominantly cited condition for mold proliferation (84%). Limited air circulation in the storage area was mentioned by 38%. Around one-quarter were aware that storage in a damp environment, e.g., in wet bags, and poultry or rodents in the storage area were risk factors for mold contamination. The knowledge of favorable conditions for mold contamination was generally higher among households belonging to the FAARM intervention group than among controls.

The study population had a fair level of knowledge of the harmful effects of mold and mycotoxin contamination. Most respondents reported the adverse effects of mold contamination of food crops, such as a change in color (71%), change in taste (67%), change in smell (52%), and reduced harvest quantities (51%). Most respondents (71%) also recognized that moldy feeds were harmful to farm animals, predominantly mentioning diarrhea (92%) as a consequence, as well as less frequently reduced weight gain and growth (10%) or reduced production of milk and eggs (6%). Over 80% of respondents knew that mold that contaminated food crops might produce toxins, and of these, over 80% knew that these toxins might remain in food even after processing or cooking. Nearly all respondents (97%) were aware that toxins produced by mold were harmful to humans. As examples, many mentioned non-specific gastrointestinal disorders, including nausea, vomiting, and stomach ache (74%). Awareness of severe long-term effects in humans was much lower, with only a third mentioning chronic liver disease and less than 10% mentioning cancer and reduced growth in children.

### 3.4. Knowledge of Preventive Practices against Mold Contamination of Crops

**Figure 4:**
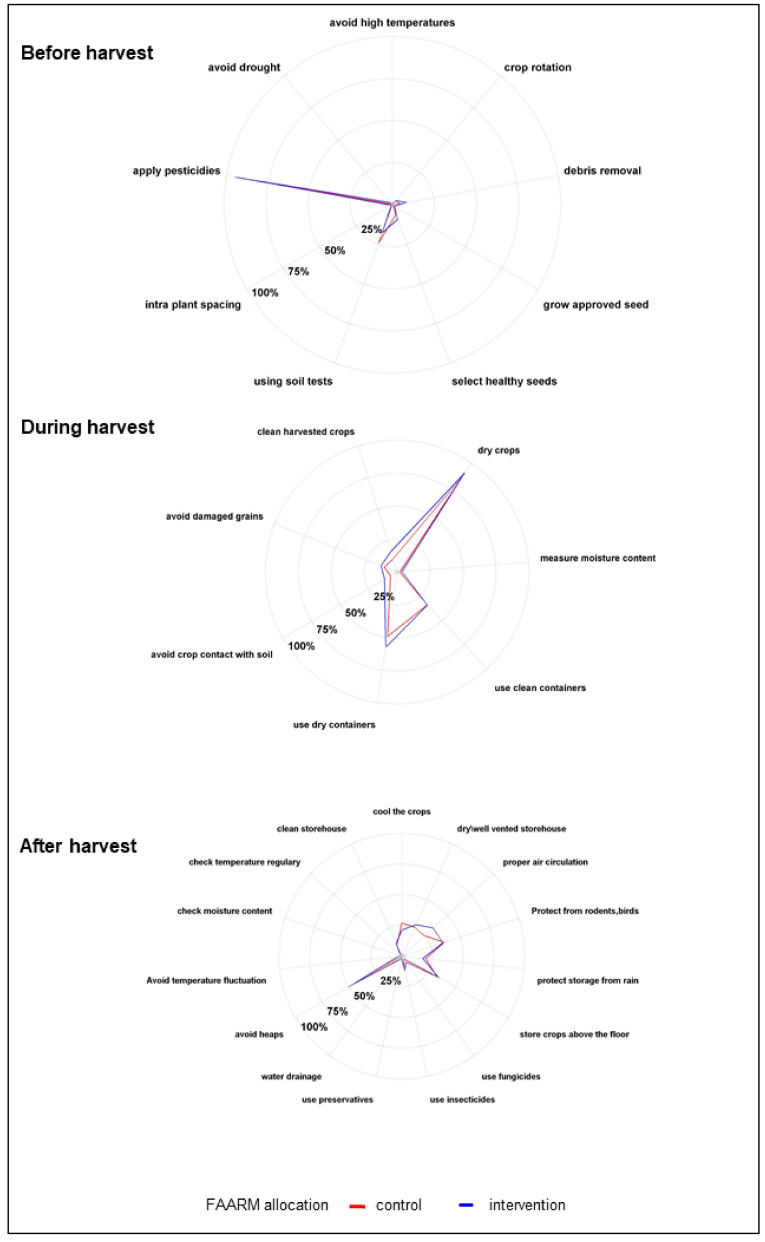
Prevention practices for mold contamination (*n* = 1280).

Figure 4 shows the commonly known preventive practices for mold contamination; in line with respondents’ knowledge of pests as favorable conditions for mold contamination, the most widely known practice for preventing mold contamination before harvest was spraying pesticides (94%). One-fifth of respondents mentioned soil tests, while other recommended practices were known by less than 10% of respondents. The predominantly known preventive practices for mold contamination during harvesting were drying crops (90%), using dry containers for harvesting (54%), and using clean containers (34%). Other recommended preventive measures at this stage, such as not collecting damaged grains and cleaning freshly harvested cereals, were known to a lesser extent. Not keeping harvested crops in heaps was the best-known preventive measure during the post-harvest and storage stages, mentioned by about half of the respondents. Around a third of respondents each knew that storing crops in a dry and well-ventilated place, maintaining air circulation, storing crops above the floor, and protecting crops from birds and rodents was protective against mold. Other recommended measures for preventing mold contamination post-harvest, such as the frequent cleaning of the storage area, were known to a lesser degree. Households in the FAARM intervention group were marginally better informed on some of these preventive measures.

**Figure 5:**
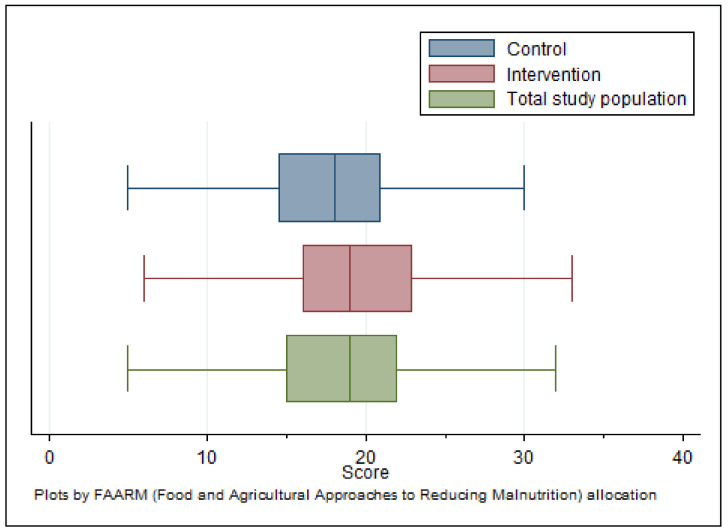
Distribution of overall knowledge score (*n* = 1280).

Figure 5 shows the distributions of mycotoxin knowledge scores for the entire study population as well as separately by FAARM allocation. The mean overall population knowledge score was 19.4 (95% CI: 18.6–20.3) out of a maximum achievable 81. FAARM intervention households achieved a mean of 20.6 and control households, 18.2 (*p* = 0.004). Based on the scoring criteria, 85% of households were classified as having a low knowledge level (<30% of maximum achievable score) regarding the timing and conditions for mold contamination of crops. In comparison, 14% had a fair level of knowledge, and only 1% had good knowledge (achieving at least 60% of the maximum score). The vast majority of households (86%) were judged to have a low level of knowledge of recommended preventive practices, while 14% had a fair knowledge level. In contrast, the knowledge of the harmful effects of mold and mycotoxin contamination of crops was much higher, with 77% of households having a fair level of knowledge, an additional 4% a good level of knowledge, and only 19% having low knowledge, in this regard. Combining all the knowledge domains, 80% of households had a low overall knowledge of mold and mycotoxin contamination and prevention. The remaining fifth had a fair level of knowledge, and less than 1% had good knowledge.

### 3.5. Socio-Demographic Characteristics and Knowledge Scores

The overall mold and mycotoxin knowledge score was associated with various socio-demographic characteristics in bivariable analyses (Supplementary Table S2).

In multivariable regression analyses (Table 2), belonging to the FAARM intervention group was linked to two additional knowledge points (compatible with 0.7 to 3.5 points more), compared to the control group. Similarly, a homestead land size of at least 30 decimals was linked to 2–3 additional knowledge points (compatible with 0.3 to 4–5 points more), and having >200 decimals of agricultural land to 1.2 extra knowledge points (compatible with >0 to 2.4 points more), compared to those with the least land. Compared to rice paddy farmers, household reliance on foreign remittances was linked to two fewer knowledge points (compatible with 0 to 4 points less), with a similar effect size but even more uncertainty if the head’s occupation was non-paddy farmer or “other”. There was no evidence for household size, head’s age, or livestock/poultry ownership being associated with the knowledge of mycotoxins when adjusting for other variables.

## 4. Discussion

In most low-income settings where mycotoxin exposure is prevalent, the knowledge, awareness, and practice of rural subsistence farmers’ regarding mold contamination of food crops remains poorly studied. This survey was thus conducted among rural Bangladeshi farming households to (i) determine their level of awareness and experience with mold contamination of food crops (ii) and to assess their knowledge of the contamination conditions and harmful effects of mold contamination, as well as (iii) their knowledge of the recommended agricultural practices for controlling and preventing mold contamination of food crops.

As far as the authors are aware, this is the first KAP study on mold and mycotoxin contamination of food crops in Bangladesh and its neighboring countries. So far, a limited number of studies worldwide have been conducted on this topic. The high general awareness of and experience with mold in this study agrees with the findings of the limited, available studies on this topic, conducted mainly in eastern and southern African countries [35,36,37,38]. The identified gaps in the knowledge of mold contamination conditions and preventive practices are, however, context-specific. Despite the research focus in an African context, mold and mycotoxin contamination of food crops is not limited to this location. For example, in the Asian sub-region, mycotoxins are reported in rice, the primary staple food [39,40].

The almost universal level of awareness of mold in general, and of mold contamination of food crops amongst respondents provides a true reflection of the ubiquitous presence of mold in the environment. The identified local terms for “mold’: “Chhatrak”, “Chita”,” Maista”, “Maiska”, and “Kalo Dag”, were spontaneously known and understood by the majority of respondents (60%). This makes these terms useful and applicable in subsequent mold and mycotoxin research in the country. Aside from the research, these local terms may also help agricultural and health officers improve information, education, and communication interventions regarding mold and mycotoxin, as they avoid the difficulties with using the technical terms. For example, in a similar study conducted in Malawi [35], only 11% and 3% of respondents were familiar with the terms “aflatoxin” and “mycotoxin,” respectively.

The predominant experience of mold contamination during the field and post-harvest stages of crop production in this community indicates a possible widespread exposure to both field mold, mainly the *fusarium* species, and storage mold, mainly the *Aspergillus* and *Penicillium* species, and their respective mycotoxins [5]. The recent biomarker surveys among different population groups in rural and urban Bangladesh [22,23,24,25,26,27,28] further support the possibility of frequent dietary exposure to multiple mycotoxins among study respondents included in the present survey.

Understandably, the respondents’ knowledge of the timing of mold contamination appears to be in keeping with their personal experience with mold contamination, mainly during the pre-harvest stage. Their responses, however, demonstrate a lack of awareness of the possibility of mold contamination during the harvesting and post-harvest storage stages of crop production. The respondents’ knowledge of favorable contamination conditions during the pre-harvest, harvesting, and storage stages of crop production appears limited. However, what they know about contamination conditions seems to be in keeping with their knowledge of the recommended agricultural practices to prevent mold contamination at the different crop production stages [21,29,40]. This relationship between knowledge and preventive practices illustrates how the appropriate information could improve knowledge and, ultimately, good agricultural practices that will help control or prevent mold contamination and subsequent exposure to mycotoxins.

In the present study, the respondents’ knowledge was relatively better regarding the observable changes in mold-contaminated crops, the awareness of subsequent mycotoxin production, and the persistence of these toxins even after processing and cooking. Similarly, the majority of the respondents recognized that mold was dangerous to both humans and livestock. Considering that these households were commonly involved in subsistence farming, these results were not surprising. They were likely to be borne from their personal experiences. Unfortunately, in low-resource settings, an awareness of the harmful effects of mycotoxins does not necessarily translate into the avoidance of moldy foodstuffs. For example, in a similar survey in Malawi, although most respondents (88%) knew that mycotoxins were harmful to human health, about half of them still consumed these contaminated foods due to a food shortage [35].

In the multivariable analysis, having the highest level of arable land was associated with a better knowledge score (Table 2). Besides having a higher farming capability and thus experience, larger farms are also more likely to be targeted by agricultural extension officers. The extension officers may educate farmers on good farming practices, the adverse effects of mold contamination of crops, and appropriate preventive practices. Likewise, the FAARM intervention’s positive impact on mold and mycotoxin knowledge could be due to transferrable skills. Although mycotoxin issues were not explicitly covered in FAARM training modules, some experienced field facilitators and supervisors involved in implementing the FAARM intervention may have covered specific mold issues as part of measures to prevent losses and improve crop yields. The finding that more respondents from FAARM intervention households had correctly explained the local term of “mold” compared to respondents from control households may indicate that FAARM field facilitators may have used these terminologies in their training. Additionally, the finding that household experience with the mold contamination of crops during the growing period appears to be less critical for FAARM intervention households than control households also suggests the beneficial effect of having marginally greater knowledge on this subject.

However, the study population’s overall low average knowledge score (19.4 out of 81) underpins an urgent need for tailored information, as well as educational and communicational intervention in this setting. To the best of our knowledge, there have been no recent interventions in farming households in the study area to raise awareness of mold contamination of farm crops and inform on the relevant preventive agricultural practices. In this study, the households who are less informed regarding mold contamination of food crops also had smaller farms and are most likely not targeted by agricultural extension officers. Nevertheless, since most households are involved in subsistence farming and food storage, at least at some point in the year, mold awareness campaigns should not ignore households less involved with farming.

This study’s unique strengths include using the appropriate local terms for mold and mycotoxins during data collection, using a probabilistic sampling approach with adequate sample size, and ensuring a high response rate to minimize bias and maximize the validity of study results. Furthermore, the design of the knowledge score was based on current evidence-based recommendations and accounted for the relative importance of different knowledge categories. The comprehensive data collected also allowed for the evaluation of the relationship between mold and mycotoxin knowledge and several household socio-demographic characteristics. Despite these strengths, one needs to take into consideration the following inherent limitations when interpreting the results. Firstly, as with most KAP studies, this study’s findings relate to reported rather than observed practices. Thus, it is assumed that the inability to provide correct answers reflects a real lack of knowledge and not a misunderstanding of the questions. Nevertheless, careful planning, pre-testing, and proper training of local data collectors were carried out to minimize any cultural or technical gaps and resolve any unclear issues.

It is also worth considering that, as many attributes are compared simultaneously between the FAARM intervention and control groups, it becomes more likely that the groups will differ on at least one attribute due to chance alone. Consequently, one needs to interpret some of the observed differences in the comparative analyses with caution. Finally, as the study was conducted in rural households in Habiganj district, who also participated in the FAARM trial, the findings cannot necessarily be generalized to other areas. However, the FAARM study population is typical for rural settings in Bangladesh and so findings may also apply to similar settings. Moreover, it may also be worthwhile collecting data on people living in Bangladesh’s urban areas to obtain a general idea about mold and mycotoxins knowledge among non-farming households. Nevertheless, the study provides valuable insights into the levels of awareness and experience, as well as the knowledge of rural farming households in Bangladesh regarding mold and mycotoxin contamination of food crops. Thus, the study provides a primary point of reference that will help the relevant public health and agricultural officers set priorities, develop practical information, educational and communicational interventions, and measure the changes from their interventions.

## 5. Conclusions

This study’s findings demonstrate that, in low-income settings with a high mycotoxin occurrence, rural farming households, who may be aware of mold contamination of food crops in general, may still have substantial knowledge gaps regarding the prevention and control of mold contamination and exposure to mycotoxins. Providing rural farming households with better information about mold control and prevention in food crops could improve their knowledge and limit exposure to mycotoxins in the community. Even though this knowledge does not necessarily lead to a change in behavior, the identified gaps in the population’s knowledge of mold contamination conditions, and the consequences of the mold contamination of crops, should form the basis for developing tailored interventions to prevent and manage mold mycotoxin contamination at different stages of crop production.

## Figures and Tables

**Table 1:** Demographic and socio-economic characteristics of the study population.

Characteristic	Overall Sample	
	*n*	%
**Religion**	1280	
Muslim		72
Hindu		28
**Household wealth quintile** ^1^	1277	
Poorest		1
Lower		11
Middle		35
Upper		40
Wealthiest		13
**Household head’s education level**	1278	
No formal education		41
Partial primary		21
Complete primary		12
Partial secondary		20
Completed secondary		4
Post-secondary		2
**Household head’s sex**	1280	
Male		96
**Household’s main income source**	1280	
Farmer (rice paddy)		28
Farmer (not paddy)		5
Unskilled day laborer		19
Skilled day laborer		5
Transport		4
Salaried/professional		5
Businessman		16
Remittances		6
Other		1
**Livestock/poultry ownership**	1280	
Yes		81
**Continuous variables (units)**		mean (95% CI)
Homestead land (decimal) ^2^	1262	11 (9–13)
Agricultural land (decimal) ^2^	1280	153 (93–213)
Household head’s age (years)	1280	43 (42–44)
Household size (persons)	1280	7 (6–7)

^1^ This is an estimate of the national wealth quintile constructed using www.equitytool.org (accessed on 10 July 2021), and not a relative wealth quintile; ^2^ A decimal is 1/100th of an acre or around 40 square meters.

**Table 2:** Adjusted associations of socio-demographic characteristics with overall mold and mycotoxin knowledge score (*n* = 1257).

Socio-Demographic Characteristics	Overall Knowledge Score		*p*-Value *
	Adjusted β	95% Confidence Interval	
Household head’s main occupation (reference = rice paddy farmer)			
No income	−1.03	−2.87, 0.82	0.28
Farmer (not paddy)	−1.81	−4.07, 0.45	0.12
Unskilled day laborer	−0.37	−1.78, 1.05	0.61
Skilled day laborer	−0.66	−3.12, 1.81	0.60
Transport	0.07	−1.96, 2.10	0.95
Salaried/Professional	0.78	−1.36, 2.91	0.48
Businessman	0.17	−1.48, 1.82	0.84
Remittances	−1.94	−3.94, 0.05	0.06
Other	−1.92	−4.48, 0.64	0.14
Own livestock/poultry ownership	0.45	−0.33, 1.22	0.26
FAARM intervention group	2.08	0.69, 3.47	0.003
Household head’s age (per year increase)	0.03	−0.01, 0.07	0.13
Homestead land size (in decimal) (reference = landless, i.e., <10)			
10–19	0.71	−0.36, 1.79	0.19
20–29	0.42	−1.50, 2.33	0.67
30–39	2.82	0.32, 5.32	0.03
≥40	2.26	0.25, 4.28	0.03
Agricultural land size (reference = <20 decimal)			
20–50	−0.09	−1.85, 1.67	0.92
51–100	0.26	−1.22, 1.74	0.73
100–200	0.27	−1.10, 1.63	0.70
>200	1.21	0.06, 2.36	0.04
Household size (reference = small, i.e., 2–4 members)			
Medium (5–10 members)	0.17	−0.55, 0.88	0.64
Large (>10 members)	−0.07	−1.90, 1.76	0.94

Mixed-effects linear regression analysis applying sampling probability weights, and adjusted for household wealth, education, and all other variables listed: β- beta coefficient; * *p*-values for Wald test; a decimal is 1/100th of an acre or around 40 square meters.

**Table 3:** Level of awareness, understanding, and experience with mold contamination.

Response	Overall Sample			by FAARM Allocation		*p*-Value *
				Control (*n* = 618)	Intervention (*n* = 662)	
	*n*	%^r^	95% Confidence Interval	%^c^	%^c^	
Familiar with local mold terms used	1280	60	(56–65)	56	64	0.08
Explained mold correctly	799	84	(80–88)	44	58	0.004
Aware that mold may contaminate crops	1280	99	(98–100)	99	100	0.13
Experienced mold contamination in crops	1273	85	(81–88)	87	82	0.17
Before harvesting	1061	82	(78–86)	75	65	0.03
During harvesting	1061	17	(14–21)	13	16	0.27
Post-harvest and storage	1061	26	(22–30)	19	25	0.06

%^r^—row percent (of *n* in the row, which may be a subgroup); %^c^—column percent (of total *n* in the column); * *p*-values for Wald test for differences between FAARM control and intervention households; FAARM—Food and Agricultural Approaches to Reducing Malnutrition trial.

**Table 4:** Knowledge of contamination conditions, harmful effects, and preventive practices regarding mold and mycotoxin contamination of food crops.

Responses	Overall Population			by FAARM Trial Allocation		*p*-Value *
				Control (*n* = 618)	Intervention (*n* = 662)	
	*n*	%^r^	95% Confidence Interval	%^c^	%^c^	
Contamination Conditions						
Timing of mold contamination	1273					
Before harvesting		88	(85–91)	89	86	0.30
During harvesting		31	(26–36)	24	36	0.01
Post-harvest/storage		38	(33–43)	31	44	0.01
Factors favoring mold proliferation before harvest	1113					
Repeated planting		1	(0.6–2.0)	0.4	1.5	0.05
Bad seeds		6	(4.4–8.7)	3.6	7.2	0.05
High temperatures		20	(16–24)	17	18	0.72
Drought		47	(42–52)	43	39	0.46
Crops attacked by pests		83	(80–87)	76	71	0.18
Field with old harvest debris		1	(0.5–1.9)	0.6	1.2	0.23
Overcrowding		1	(0.4–1.6)	0.0	1.4	0.01
Overgrown weeds		18	(15–22)	11	21	<0.001
Other (mainly lack of vitamin/fertilizer and flooding)		15	(12–18)	13	13	0.94
Factors favoring mold proliferation during harvest	376					
Dirty containers		18	(12–26)	3.2	7.7	0.04
Moist containers		48	(41–56)	8	21	0.001
Moldy containers		16	(11–24)	2.3	7.5	0.01
Insect-infested containers		13	(9–20)	2.7	5.4	0.11
Damaged grains		8	(5–14)	0.7	4.2	0.01
Soil left on pods		27	(22–33)	5	11	0.02
Leaving in damaged grains		5	(2.8–9.2)	0.6	2.5	0.05
High levels of rain		79	(73–84)	19	29	0.03
Delayed/harvesting		16	(12–21)	3.1	6.8	0.03
Other (mainly delayed threshing)		1	(0.4–4.9)	0.3	0.5	0.68
Factors favoring mold proliferation after harvest	467					
Heaping wet, freshly harvested crops		84	(79–88)	25	38	0.01
Limited air circulation		38	(32–44)	10	18	0.002
Storing without rain protecting		14	(11–18)	4.1	6.4	0.12
Storing without drainage		7	(4.3–11.5)	1.1	4.2	0.03
Storing in wet environment		28	(23–33)	8	13	0.10
Rodents/birds in storage area		29	(22–36)	9	13	0.22
Storing in wet bags		26	(20–32)	7	12	0.11
Storing bags on the floor		7	(4.2–10.0)	1.1	3.8	0.02
Other (mainly insect attack, delayed & threshing)		1	(0.6–3.4)	0.5	0.6	0.84
Harmful effects of mold						
Effect of mold contamination of crops	1273					
Change in color		71	(66–74)	65	75	0.011
Change in taste		67	(63–72)	59	74	<0.001
Change in smell		52	(48–57)	48	56	0.04
Reduced harvest quantities		51	(46–55)	53	48	0.224
Reduced market value		33	(28–38)	31	35	0.470
Harmful effects of moldy feeds on animals	1273	71	(67–74)	69	72	0.43
Reduces production of milk or eggs	903	6	(3.6–11.4)	1.7	7.3	0.03
Reduces weight gain and growth	903	10	(7–15)	5.6	8.6	0.31
Causes fever	903	5	(3.3–6.9)	2.5	4.2	0.16
Causes death	903	16	(13–19)	13	10.0	0.18
Causes diarrhea	903	92	(90–94)	63	67	0.21
Others (mainly other gastro-intestinal problems)	903	18	(14–22)	13	13	0.98
Mold that contaminates crops and produces toxins	1273	84	(80–87)	83	84	0.88
Mold toxins may persist in processed or cooked food	1052	88	(84–91)	75	73	0.68
Harmful effects of mycotoxin on humans	1052	97	(95–98)	80	82	0.72
Chronic liver diseases	1018	30	(25–36)	19	30	0.02
Reduced child growth	1018	2	(0.8–3.8)	0.8	2.1	0.26
Cancers	1018	7	(4.6–10.9)	4.6	6.9	0.36
Others (mainly gastro-intestinal problems)	1018	74	(69–79)	62	58	0.50
Preventive practices						
Preventing mold contamination before harvest	1273					
Crop rotation schedule		3	(2.0–4.9)	2.9	3.3	0.771
Plowing under/removing debris		7	(4.4–9.9)	4.5	8.6	0.127
Using soil tests		21	(17–25)	24	18	0.080
Select healthy seeds		8	(5.2–11.3)	6.1	9.2	0.296
Growing recommended seed		1	(0.5–1.8)	0.5	1.5	0.091
Timing planting to avoid high temperatures		1	(0.2–1.1)	0.6	0.4	0.625
Timing planting to avoid drought stress		1	(0.7–2.3)	0.6	1.9	0.060
Maintaining intra-plant spacing		0.4	(0.1–1.5)	0.0	0.7	0.170
Spraying pesticides		94	(92–96)	93	95	0.393
Other (mainly applying fertilizer, vitamins, or charcoal/ash)		9	(6.6–11.4)	9.1	8.3	0.761
Preventing mold contamination during harvest	1273					
Using clean containers		34	(30–39)	34	34	0.97
Using dry containers		54	(49–58)	49	58	0.06
Not collecting damaged grains		12	(9–15)	10	13	0.32
Avoiding contact with soil		8	(6.7–10.5)	6	11	0.01
Measuring moisture content		3	(2.0–4.5)	2.1	3.8	0.20
Drying the crop		90	(87–92)	87	91	0.15
Cleaning freshly harvested cereal		13	(11–17)	10	16	0.07
Other (mainly prompt threshing and covering with plastic)		1	(0.3–1.4)	0.6	0.6	0.85
Preventing mold contamination after harvest	1273					
Not keeping in heaps		48	(43–53)	45	50	0.34
Maintaining air circulation		30	(25–35)	25	34	0.04
Storing in dry/well-ventilated structure		27	(23–32)	26	28	0.64
Storing protected from rain		18	(15–22)	20	17	0.52
Storing in an area with water drainage		4	(2.5–5.3)	4.0	3.3	0.64
Storing protected from rodents/birds		36	(31–41)	36	35	0.84
Protecting storage area from temperature fluctuation		3	(1.8–4.3)	1.3	4.1	0.02
Cooling the crops		24	(19–30)	27	21	0.30
Storing crops above the floor		34	(29–39)	33	35	0.72
Checking moisture content regularly		1	(0.4–1.6)	0.9	0.8	0.78
Checking temperatures regularly		1	(0.7–2.2)	1.0	1.5	0.42
Using insecticides		11	(8–14)	10	12	0.47
Using fungicides		5	(2.9–7.0)	2.8	6.2	0.08
Cleaning storage area frequently		11	(8–14)	10	11	0.59
Using preservatives		2	(0.9–2.6)	1.5	1.6	0.89
Other (mainly applying Neem leaf powder and catching insects)		2	(1.0–2.8)	0.9	2.3	0.10
Knowledge of contamination conditions	1280					
Low (<30 of max. score)		85	(81–88)	88	82	0.13
Fair (30–59)		14	(11–17)	12	15	0.29
Good (60+)		1	(0.7–2.5)	0.2	2.3	0.01
Knowledge of harmful effects						
Low (<30 of max. score)		19	(16–23)	20	18	0.47
Fair (30–59)		77	(73–81)	79	76	0.43
Good (60+)		4	(2.3–6.9)	1.1	6.7	0.01
Knowledge of preventive practices						
Low (<30 of max. score)		86	(81–89)	88	84	0.28
Fair (30–59)		14	(11–19)	12	16	0.28
Overall knowledge level	1280					
Low (<30 of max. score)		80	(76–84)	85	76	0.02
Fair (30–59)		19	(16–24)	15	24	0.02
Good (60+)		0.2	(0.1–0.9)	0.0	0.5	0.14
Mean knowledge scores	1280	Mean				
Contamination conditions		5.7	(5.3–6.1)	5.1	6.3	0.002
Harmful effects		7.7	(7.4–8.0)	7.4	8.0	0.03
Preventive practices		6.0	(5.7–6.3)	5.7	6.3	0.08
Overall knowledge		19.4	(19–20)	18	21	0.004

%^r^—row percent (of *n* in the row, which may be a subgroup); %^c^—column percent (of total *n* in the column); * *p*-values for Wald test for differences between FAARM control and intervention households; FAARM—Food and Agricultural Approaches to Reducing Malnutrition trial.

## Data Availability

The data presented in this study are available upon reasonable request from the corresponding author. The data are not publicly available due to privacy restrictions.

## Supplementary Materials

The following are available online at https://www.mdpi.com/article/10.3390/ijerph181910335/s1, Table S1a, and, Table S1b: Mold and mycotoxin knowledge questions, scoring criteria, and categorization. Table S2: Crude associations of socio-demographic characteristics with overall knowledge.
