# Supplementary material for: Awareness, Experience, and Knowledge of Farming Households in Rural Bangladesh Regarding Mold Contamination of Food Crops: A Cross-Sectional Study
Source: Int J Environ Res Public Health. 2021 Sep 30;18(19):10335. doi: 10.3390/ijerph181910335 (PMC8508072; doi:10.3390/ijerph181910335)
Supplement: Supplementary file 1 [file ijerph-18-10335-s001.zip › ijerph-1315195-supplementary.pdf]

Supplementary Table S1a: Mold and mycotoxin knowledge questions and scoring criteria.

Question		Response	Scoring	Total
Contamination and conditions				
k 1	Can molds ("Chhatrak" or "Chita" or" Maista" or "Maiska" or "Kalo Dag") be present in farm crops?	No	0	1
		Yes	1	
		Don't know	0	
k 2	At what stage of crop production can mold contamination occur?	Before harvesting	1	3
		During harvesting	1	
		Post-harvest and storage	1	
		Don't Know	0	
k 3	Can you tell me of any conditions before harvesting that may promote mold contamination?	None mentioned	0	8
		Repeated planting of the same crop on the same plot	1	
		Bad or inappropriate seeds	1	
		High temperatures	1	
		Drought	1	
		Crops attacked by pests.	1	
		Sowing in a field that still has old harvest debris	1	
		Overcrowding due to poor inter-plant spacing	1	
		Too many weeds in crops	1	
		Doesn't know	0	
k 4	Can you tell me of any conditions during harvesting that may promote mold contamination?	None mentioned	0	9
		Harvesting with dirty containers	1	
		Harvesting with moist containers	1	
		Harvesting with moldy containers	1	
		Harvesting with insect-infested containers	1	
		Damaged grain or produce	1	

		When there is soil left on pods after harvesting	1	
		Leaving in damaged grains / foreign matter from harvested crops	1	
		High levels of rain during the harvesting period	1	
		Delayed or late harvesting	1	
		Doesn't know	0	
k5	Can you tell me of any conditions during post-harvest and storage that may promote mold contamination?	None mentioned	0	8
		Heaping wet, freshly harvested crops for long periods	1	
		Limited circulation of air through the storage area	1	
		Storing grains without protection from rain or groundwater	1	
		Storing grains without drainage	1	
		Storing grains in a wet environment	1	
		Rodents, poultry, or other birds in the storage area	1	
		Storing grains in wet and/or unclean bags	1	
		Storing bags of grains directly on the floor	1	
		Doesn't know	0	
Harmful effects of mold and mycotoxin contamination				
k6	How does mold contamination affect farm crops?	No changes	0	5
		Crops change in color	1	
		Crops change in taste	1	
		Crops change in smell	1	
		Reduced harvest quantities	1	
		Reduced market value	1	
		Don't know	0	
k7	Are there any harmful effects of giving farm animals feed prepared from moldy grains?	No	0	1
		Yes	1	
		Don't know	0	
	What are some of these harmful effects in animals?	Reduces production of milk or eggs	1	7

k 8		Reduces weight gain and growth	1	
		Causes fever	1	
		Causes death	1	
		causes diarrhea	1	
		reduced feeding	1	
		Other gastrointestinal disturbances	1	
		None mentioned	0	
k 9	Can molds that contaminate rice and other staple crops produce some toxins?	No	0	1
		Yes	1	
		Don't know	0	
k 10	Can these toxins still be present in prepared foods on the table after harvest?	No	0	1
		Yes	1	
		Don't know	0	
k 11	Are there any harmful effects of toxins on humans?	No	0	1
		Yes	1	
		Don't know	0	
k 12	Can you name any of these harmful effects on humans?	Chronic liver diseases	1	5
		Reduced growth of children	1	
		causes diarrhea	1	
		Other gastrointestinal disturbances	1	
		Cancers	1	
		None mentioned	0	
Preventive practices				
k 13	Can you tell me anything you do before harvesting to reduce mold contamination?	Nothing	0	9
		Maintaining a crop rotation schedule	1	
		Preparing the seedbed by plowing under or removing debris	1	
		Using soil tests to determine fertilizer/soil conditioner needs	1	
		Select healthy seeds	1	
		Growing recommended seed varieties for my farm area	1	

		Timing crop planting to avoid high temperature	1	
		Timing crop planting to avoid drought stress during the period of seed development and maturation	1	
		Maintaining the recommended row and intra-plant spacing.	1	
		Spraying pesticides	1	
		Doesn't know	0	
k 14	Can you tell me anything you do during harvesting to reduce mold contamination?	Nothing	0	7
		Harvesting with clean containers	1	
		Harvesting with dry containers	1	
		Not collecting grains that are damaged	1	
		Avoiding contact of the grain with soil	1	
		Measuring the moisture content of harvested grain	1	
		Drying the crop immediately after harvest.	1	
		Cleaning freshly harvested cereals to remove damaged kernels and other foreign matter.	1	
		Don’t know	0	
k 15	Can you tell me of any measure you implement during post-harvest and storage to prevent mold contamination?	Nothing	0	15
		Not keeping freshly harvested commodities in heaps for more than a few hours before drying or threshing	1	
		Maintaining air circulation through the grain storage area	1	
		Storing grains in a dry, well-vented structure	1	
		Storing grains protected from rain	1	
		Storing grains in an area with water drainage	1	
		Storing grains protected from rodents/birds	1	
		Protecting the grain storage area from temperature fluctuations	1	
		Cooling the crops as quickly as possible after drying	1	
		Storing crops above the floor or with a water-impermeable layer	1	
		Checking moisture content of grain at regular intervals	1	
		Checking the temperature of stored grain at regular intervals	1	

		Using insecticides	1	
		Using fungicides	1	
		Cleaning the storage area frequently	1	
		Using preservatives	1	
		Don’t know	0	
		**Total maximum score**		**81**

Supplementary Table S1b: Knowledge level categorization.

**Knowledge category**	**Max score**	**Low**	**Fair**	**Good**
Contamination and conditions	29.0	0-9	9-17	≥18
Harmful effects	21.0	0-6	6-11	≥12
Preventive practices	31.0	0-9	9-18	≥19
Total maximum score	**81.0**	0-23	24-47	≥48
Criteria for categorizing knowledge level				
Low knowledge (less than 30%)				
Fair knowledge (30-59%)				
Good (≥60%)				

Supplementary Table S2: Crude associations of socio-demographic characteristics with overall knowledge.

Socio-demographic characteristics	N	Overall knowledge score		P-value*
		Crude β	95% CI	
Hindu religion (reference= Muslim)	1280	-0.79	-2.81, 1.23	0.44
Household wealth (reference = lowest two quintiles)	1277			
middle quintile		0.68	-0.64, 2.01	0.31
upper quintile		0.83	-0.60, 2.25	0.25
wealthiest quintile		1.17	-0.44, 2.78	0.15
Household head's education (reference = no education)	1278			
Partial primary		-0.56	-1.56, 0.43	0.27
Complete primary		0.41	-0.93, 1.75	0.55
Partial secondary		-0.16	-1.36, 1.05	0.80
Complete secondary or more		1.22	-0.74, 3.18	0.22
Household head is female (reference = male)	1280	-1.37	-3.46, 0.71	0.20
Household head's main occupation (reference = rice farmer)				
No income	1280	-0.07	-1.62, 1.49	0.93
Farmer (not paddy)		-2.05	-4.37, 0.28	0.08
Unskilled day laborer		-1.35	-2.55, -0.14	0.03
Skilled day laborer		-1.81	-3.87, 0.25	0.09
Transport		-0.78	-2.67, 1.11	0.42
Salaried/Professional		0.19	-1.77, 2.15	0.85
Businessman		-0.34	-1.74, 1.06	0.63
Remittances		-2.32	-4.11, -0.52	0.01
Other		-2.58	-4.89, -0.27	0.03
Own livestock/poultry ownership	1280	0.85	0.06, 1.63	0.03
FAARM intervention group (vs. control)	1280	2.18	0.84, 3.52	0.001
Household head's age (per year older)	1280	0.04	0.01, 0.07	0.01

Homestead land size reference= <10 decimal	1262			
10 - 19		1.08	0.15, 2.02	0.02
20 - 29		0.90	-0.77, 2.57	0.29
30 - 39		3.62	1.08, 6.15	0.01
≥ 40		3.22	1.32, 5.12	0.001
Agricultural land size (reference= <20 decimal)				
20-50	1280	0.51	-1.18, 2.21	0.55
51-100		0.76	-0.61, 2.14	0.28
100-200		0.78	-0.40, 1.96	0.20
> 200		2.02)	1.14, 2.91	<0.001
Household size	1280	0.26	0.13, 0.40	
(reference= Small i.e. 2-4 members)				
Medium (5-10 members)		1.025	0.29, 1.76	0.01
Large (>10 members)		2.016	0.43, 3.61	0.01

Mixed-effects bivariable linear regression applying sampling probability weights; β- beta coefficient;
95% CI- 95% confidence interval; *p-values for Wald test; A decimal is 1/100th of an acre or around 40 square meters
